# Plasmapheresis for treatment of immune complex-mediated glomerulonephritis in infective endocarditis: a case report and literature review 

**DOI:** 10.5414/CNCS109082

**Published:** 2017-04-13

**Authors:** Meredith Halpin, Olga Kozyreva, Vanesa Bijol, Bertrand L. Jaber

**Affiliations:** 1 Department of Medicine, Division of Nephrology, St. Elizabeth’s Medical Center,; 2 Department of Medicine, Tufts University School of Medicine,; 3 Department of Medicine, Division of Hematology & Oncology, St. Elizabeth’s Medical Center,; 4 Department of Pathology, Brigham and Women’s Hospital, and; 5 Harvard Medical School, Boston, MA, USA

**Keywords:** subacute endocarditis, Streptococcus mutans, immune complex, proliferative glomerulonephritis, plasma exchange

## Abstract

We report the case of a 57-year-old man who presented with subacute bacterial endocarditis secondary to *Streptococcus mutans* complicated by biopsy-proven immune complex-mediated glomerulonephritis (ICGN). Despite initial treatment with antibiotics and a short course of corticosteroids, the kidney function further deteriorated, and plasmapheresis was introduced as third-line therapy to remove circulating immune complexes. Following 7 treatment sessions, the patient recovered kidney function. We discuss the potential merit of plasmapheresis for patients with subacute bacterial endocarditis who develop ICGN.

## Introduction 

Immune complex-mediated glomerulonephritis (ICGN) is a known complication of infective endocarditis, in particular subacute bacterial endocarditis [1, 2, 3]. Infections with less virulent bacterial organisms, by virtue of their indolent subacute course, promote an antibody response that predisposes to the formation of immune complexes and the development of glomerulonephritis. In patients with subacute bacterial endocarditis, circulating immune complexes deposit on the glomerular basement membrane and activate the complement system, leading to an immune response and glomerular injury [1, 4, 5]. Control of the infection generally leads to resolution of the glomerular inflammation and normalization of kidney function. The use of plasmapheresis as salvage therapy for patients with bacterial endocarditis-associated ICGN is controversial [6]. 

We present the case of a 57-year-old man who presented with biopsy-proven ICGN in the setting of bacterial endocarditis secondary to *Streptococcus mutans*. Despite conservative management, and in light of worsening kidney function and elevated levels of circulating immune complexes, he was empirically treated with plasmapheresis, which was followed by improvement in kidney function. We review the existing literature on the potential role of plasmapheresis as an adjunct to the treatment of ICGN in the setting of bacterial endocarditis. 

## Case report 

A 57-year-old man with no significant medical history presented to an outside hospital with symptoms of malaise, a 30-pound weight loss, arthralgias, and night sweats of 2-month duration. He was diagnosed with subacute bacterial endocarditis on the basis of *Streptococcus mutans* bacteremia and echocardiographic evidence of vegetations on the tricuspid and mitral valves. After 12 days, he was transferred to our hospital for further evaluation and management. Upon presentation, he had evidence of multiple organ system involvement, including acute kidney injury, septic emboli to the lungs, central nervous system embolic lesions, and a petechial rash over the lower extremities. He was treated with intravenous ceftriaxone 2 g daily with rapid resolution of the bacteremia. His baseline kidney function from a prior year was normal with a serum creatinine of 1.0 mg/dL. On presentation, he reported cola-colored urine, and was found to have acute kidney injury with a serum creatinine of 3.0 mg/dL. The urinalysis revealed 2+ protein and large blood, and the urine sediment dysmorphic red blood cells and red blood cellular casts. Levels of complement factors C3 and C4 were low at 24 mg/dL (normal range, 75 – 175 mg/dL) and 5 mg/dL (normal range, 14 – 40 mg/dL), respectively. The ANA screen was positive with a titer of 1 : 160, but the antimyeloperoxidase, antiproteinase 3, anti-GBM, and anti-DS DNA antibody titers were nonreactive. The rheumatoid factor was present but the cyclic citrullinated peptide antibody was negative. The serum protein electrophoresis detected no monoclonal proteins. 

**Figure 1.**
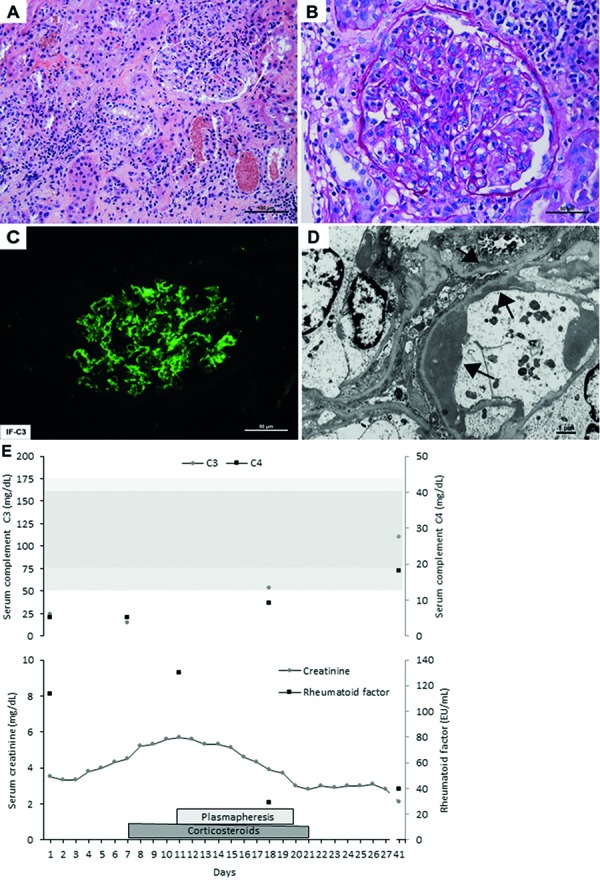
Kidney biopsy findings of immune complex-mediated glomerulonephritis and time course of disease. A: A hypercellular glomerulus, interstitial inflammation, and red blood cell casts are seen on intermediate-power light microscopy (PAS stain, 100×). B: Global endocapillary proliferation without crescent formation is seen on high-power light microscopy (PAS stain, 400×). C: Significant reactivity for C3 (the strongest of all components) is seen on direct immunofluorescence microscopy (400×). D: Large sub-endothelial deposits (labeled with arrows) are seen on electron microscopy. E: Time course of the serum creatinine, rheumatoid factor, and complement component C3 and C4 over the duration of the hospitalization.

On hospital day 4, he underwent a percutaneous kidney biopsy, which revealed diffuse proliferative glomerulonephritis with global involvement of glomeruli and evidence of endocapillary and mesangial proliferation (Figure 1A – D). The immunofluorescence microscopy revealed heavy granular deposits of IgG, C3, and C1q as well as some IgM in the mesangium and along the glomerular basement membranes, consistent with ICGN. The patient was treated empirically with a short course of corticosteroids, consisting of pulse intravenous methylprednisolone (dose of 250 – 500 mg) for 5 days followed by an oral prednisone (starting dose of 60 mg) taper over 14 days. 

Despite appropriate antibiotics and a short course of corticosteroids, the patient’s kidney function continued to worsen and the serum creatinine rose to 5.7 mg/dL. On hospital day 11, in light of the presence of circulating immune complex, as detected by the C1q binding assay C1q level of 63 EU/mL (< 20 EU/mL), and a rheumatoid factor level of > 130 IU/mL (< 14 IU/mL), as well as persistent hypocomplementemia, he was started on an empirical course of plasmapheresis. During each session, 4.5 L of plasma were removed, and 50% was replaced with fresh frozen plasma, and 50% with albumin. After the completion of 7 sessions, there was an improvement in his kidney function, coinciding with a marked decline in the rheumatoid factor level to 39 IU/mL and near normalization of serum complement levels (C3 level 53 mg/dL and C4 level 9 mg/dL). He was discharged on hospital day 27 with a serum creatinine of 2.8 mg/dL (Figure 1E), and 13 months after resolution of the bacterial endocarditis and resolution of the glomerular inflammation, his kidney function had fully normalized, with a return of the serum creatinine to 0.8 mg/dL. 

## Discussion 

The clinical and morphological features of acute glomerulonephritis that develops during the course of bacterial endocarditis have extensively been characterized [1, 4, 7]. Post-mortem kidney biopsies obtained from patients with bacterial endocarditis during the period of 1965 – 1979 reveal an incidence rate of acute glomerulonephritis of 22.4% [7]. However, its incidence rate in the setting of acute and subacute bacterial endocarditis in the modern era of medicine with improved access to medical care and widespread use of antibiotics is less clear. In a more recent period covering 1981 – 1998, on kidney histology available from both biopsies and necropsies from patients with confirmed bacterial endocarditis, the most commonly described kidney lesion was localized renal infarcts (31% noted only on autopsy and primarily observed in patients infected with *Staphylococcus aureus*), followed by ICGN (26%), acute interstitial nephritis (10%, attributed to antibiotics), and cortical necrosis (10%) [5]. 

The typical histological expression of subacute bacterial endocarditis in the kidneys is a proliferative glomerulonephritis as a result of formation of circulating immune complexes that activate the complement system by the classic pathway, deposit in the glomeruli in the process of glomerular filtration, and elicit an inflammatory reaction. 

Our case describes a man with previously normal kidney function who presented with acute kidney injury and biopsy-proven ICGN in the setting of subacute bacterial endocarditis due to *Streptococcus mutans*. Despite appropriate antibiotic therapy and resolution of the bacteremia, his kidney function further declined. Given the lack of response to an empirical course of corticosteroids, he was initiated on plasmapheresis in light of the presence of circulating immune complexes, as detected by the C1q binding assay and rheumatoid factor level. After 7 sessions of plasmapheresis, the serum creatinine greatly improved, and more than a year later, the kidney function normalized. 

The premise of plasmapheresis is the removal of a pathologic substance from the circulation that is causing damage, and by removing the unwanted substance (e.g., a circulating auto-antibody, immune complex, cryoglobulin, or a monoclonal light chain), this can prevent or attenuate further tissue deposition and damage. The American Society for Apheresis has published guidelines on the indications for plasmapheresis [6]. While a category-1 indication would be for a disorder where plasmapheresis is accepted as first-line therapy, either as a primary standalone treatment or in conjunction with other modes of treatment, a category-4 indication would be for a disorder where the plasmapheresis might be ineffective or harmful. The potential role for plasmapheresis for ICGN falls under a category-3 indication, where the optimum role of plasmapheresis therapy has not been established and decision making should be individualized. In bacterial endocarditis, it can be hypothesized that circulating immune complexes are pathogenic, and their removal by plasmapheresis would attenuate immune deposits in the glomeruli, thereby limiting kidney injury. 

A review of the literature identifies several case reports of ICGN in the setting of bacterial endocarditis, initially treated with appropriate antibiotics but where second-line therapy with a short course of corticosteroids [8, 9] and even cytotoxic drugs, such as cyclophosphamide [10] and azathioprine [11, 12], was added to assist in the recovery of kidney function. 

The potential role of plasmapheresis as an adjunct or third-line therapy for the treatment of ICGN in the setting of bacterial endocarditis has received less attention and originates from very few published case reports. Table 1 summarizes the 5 published cases (including our case) where plasmapheresis was successfully used for this indication [11, 13, 14, 15]. All 5 patients presented with subacute endocarditis associated with a *streptococcus* species, although in 1 case the incriminated bacterium was not reported by the authors but the patient had undergone dental extraction weeks prior to presentation [13]. Four of the 5 patients were men, and the age ranged from 25 to 57 years old. All patients presented with acute kidney injury, and 2 required hemodialysis at the initial presentation due to kidney failure [13, 15]. The urinalysis revealed hematuria and proteinuria, and serum complement levels were low. The rheumatoid factor, assayed in 3 cases, was present [11, 14], and circulating immune complexes, as detected by the C1q binding assay, were present in 4 cases [11, 13, 14]. In 4 of the 5 patients, the kidney biopsy revealed fibrocellular crescentic glomerulonephritis on light microscopy, contrasting with our case where diffuse endocapillary and mesangial proliferative glomerulonephritis was observed. Immunofluorescence microscopy depicted granular glomerular immune deposits of complement components and immunoglobulins in 4 cases [11, 13, 14], whereas the 5^th^ case described a pauci-immune glomerulonephritis with nonreactive circulating ANCA and anti-GBM antibody titers [15]. Corticosteroids were used in 4 patients [11, 13, 14], and azathioprine in 1 patient [11]. Plasmapheresis was initiated at an average of 14 days from initial presentation (range, 5 – 27 days), 2 – 4.5 L of plasma were exchanged per session and replaced with either albumin or a combination of albumin and fresh frozen plasma, and patients received an average of 8 sessions (range, 5 – 14). Following plasmapheresis, the kidney function improved in all 5 patients, and there was a documented parallel decrease in levels of circulating immune complexes, as detected by the C1q binding assay in 3 patients where this marker was trended [11, 13, 14]. In our patient, the initial C1q level was markedly elevated but it was not repeated following plasmapheresis to document its removal, although the treatment coincided with improvement in the kidney function. In our case however, the rheumatoid factor level dropped significantly, which has been shown to correlate with levels of circulating immune complexes in patients with endocarditis [16]. In one study, rheumatoid factor was present in 24% of intravenous drug users presenting with endocarditis due to *Staphylococcus aureus* [17], and elevated rheumatoid factor levels in endocarditis may be part of a host-induced antibody response to elevated levels of circulating immune complexes [16]. While the observed improvement in kidney function is coincidental, we can only speculate that plasmapheresis might have shortened the natural history of the glomerulonephritis by promoting the removal of circulating immune complexes, and accelerating the resolution of the glomerular inflammation. 

When considering plasmapheresis for endocarditis-associated ICGN where its role has not been established, potential risks of the therapy need to be considered, including hypotension, removal of antibiotics, immune suppression, and bleeding; and as a result, decision making should be carefully individualized. 

Three of the 5 patients ultimately required cardiac surgery [13, 14, 15]. A repeat kidney biopsy was performed in 2 patients [13, 14], one performed 2 months later showing sclerosis in 17% of the glomeruli and old crescents in 13% of the glomeruli, and no deposits on immunofluorescence microscopy [14], and one performed 125 days after initiation of plasmapheresis, revealing sclerosing focal segmental glomerulonephritis [13]. At the end of the follow-up period, which ranged from 43 days to 2 years, all 5 patients were alive, and the average serum creatinine was 1.7 mg/dL (range, 0.8 – 2.7 mg/dL). 

In conclusion, we report on the successful use of plasmapheresis for the removal of circulating immune complexes in a patient who presented with subacute bacterial endocarditis complicated by acute kidney injury due to ICGN, and who had initially failed to respond to first- and second-line therapy with antibiotics and a short course of corticosteroids. The prognosis of ICGN in patients with bacterial endocarditis is usually good, and is related to the prompt eradication of the infection, using appropriate antibiotics for 4 – 6 weeks [18]. While there is equipoise on the role of plasmapheresis for ICGN, the observed favorable renal outcome raises the hypothesis as to whether plasmapheresis has a potential role as adjunctive therapy for the treatment of this kidney-related complication of endocarditis. Future studies are needed to assess the merit as well as the efficacy and safety of plasmapheresis for the removal of circulating immune complexes in patients who present with bacterial endocarditis complicated by ICGN. 

## Support 

There was no support/funding for this report. 

## Conflict of interest 

There were no potential conflicts of interest noted. 

**Table 1.** Summary of the clinical, laboratory, and pathology features of patients with acute glomerulonephritis in the setting of bacterial endocarditis treated by plasmapheresis (including current case report).

	McKenzie et al, 1979 [13]	Rovzar et al. 1986 [11]	Daimon et al. 1998 [14]	Couzi et al., 2004 [15]	Halpin et al. 2016 (current report)
Age (years)/sex	53/M	25/M	50/F	58/M	57/M
Urinalysis	Not reported	Hematuria, proteinuria, red and white blood cell casts	Hematuria, proteinuria	Hematuria, proteinuria (1.61 gm/24 h)	Hematuria, proteinuria, red blood cell casts
Initial serum creatinine	10.4 mg/dL	3.9 mg/dL	2.0 mg/dL	13.8 mg/dL	3.0 mg/dL
C3/C4 levels	Low/low	Low/low	Low/low	Low/normal	Low/low
Serological workup	Nonreactive anti-GBM antibody	Nonreactive ANA, ANCA, anti-GBM antibody, and cryoglobulin Elevated rheumatoid factor titer (1 : 640)	Nonreactive ANA, anti-DS-DNA antibody, ANCA, and anti-GBM antibody Elevated IgG and IgA levels; elevated rheumatoid factor level (56 IU/mL)	Nonreactive ANA, anti-DS-DNA antibody, ASO, ANCA, anti-GBM antibody, and cryoglobulin	Nonreactive anti-DS-DNA antibody, ANCA, anti-GBM antibody, and cryoglobulin Elevated ANA titer (1 : 160), elevated RF level (> 130 IU/mL)
C1q level*	Elevated	Elevated	Elevated	Undetected	Elevated
Blood culture	Not reported (dental work)	*Streptococcus viridans*	*Streptococcus viridans*	*Streptococcus parasanguis*	*Streptotoccus mutans*
Echocardiographic location of vegetations	Aortic valve	Aortic valve	Tricuspid valve	Mitral and aortic valve	Tricuspid and mitral valve
Kidney biopsy findings	LM: Focal segmental crescentic glomerulonephritis (60% of glomeruli) IF: C3 and IgM granular deposits	LM: Fibrocellular crescentic glomerulonephritis (50% of glomeruli) IF: C3, IgG, and IgM granular deposits	LM: Fibrocellular crescentic glomerulonephritis (64% of glomeruli) IF: C3 and C1q granular deposits	LM: Fibrocellular crescentic glomerulonephritis (50% of glomeruli) IF: No complement or immunoglobulin deposits	LM: Diffuse proliferative glomerulonephritis (endocapillary and mesangial proliferation) IF: C3, C1q, IgG, and IgM granular deposits
Antibiotics	Cloxacillin	Penicillin and gentamicin (initiated on day 2)	Piperacillin, amoxicillin, cefazolin	Ceftriaxone and vancomycin (initiated on day 1)	Ceftriaxone
Immunosuppressive drugs	Prednisolone	Prednisone 2 mg/kg/day and azathioprine 1 mg/kg/day (initiated on day 16 due to rising serum creatinine)	None	Prednisone 1 mg/kg/day (initiated on day 13)	Methylprednisolone 250 – 500 mg initiated on day 4 for 5 days followed by prednisone 1 mg/kg/day tapered over 14 days
Hemodialysis	Initiated on day –1	Not required	Not required	Initiated on day 1 and discontinued on day 29	Not required
Plasmapheresis	Initiated on day –8 for a total of 5 sessions; 2-L exchange using 5% albumin	Initiated on day 5 and 6 and then thrice weekly for a total of 11 sessions; 3-L exchange per (90-minute) session using 5% salt-poor albumin	Initiated on day 20 for a total of 5 sessions; 3-L exchange per session	Initiated on day 27 and discontinued on day 65 for a total of 14 sessions; 3-L exchange per session with albumin	Initiated on day 11 for a total of 7 sessions; 4.5-L exchange per session with 50% albumin and 50% fresh frozen plasma
Cardiac surgery	Aortic valve replacement on day –75	Not required	Fistula closure and vegetation excision on day 60	Valvular replacement on day 90	Not required
Duration of follow-up	132 days	43 days	10 months	2 years	13 months
Last serum creatinine	1.8 mg/dL	1.8 mg/dL	1.3 mg/dL	2.7 mg/dL	0.8 mg/dL

M = male; F = female; LM = light microscopy; IF = immunofluorescence; *The C1q binding assay measures circulating immune complexes. Note: Conversion factor for creatinine in mg/dL to mmol/L, multiply by 88.4.
